# Physiotherapists’ use of outcome measures in the assessment of lateral elbow tendinopathy: An international online survey

**DOI:** 10.1177/17585732261440542

**Published:** 2026-04-22

**Authors:** Luke Heales, Leanne Bisset, Bill Vicenzino, Marcus Bateman, Caitlin Hill, Crystal Kean, Steven Obst

**Affiliations:** 1 School of Health, Medical and Applied Sciences, Musculoskeletal Health and Rehabilitation Research Group, College of Health Sciences, 6939 Central Queensland University, Rockhampton & Bundaberg, QLD, Australia; 2 Australian Centre for Precision Health and Technology, 5723 Griffith University, Gold Coast, QLD, Australia; 3 Department of Physiotherapy, School of Health and Rehabilitation Sciences, 1974 University of Queensland, Brisbane, QLD, Australia; 4 Physiotherapy/Orthopaedics, 2102 University Hospitals of Derby & Burton NHS Foundation Trust, Derby, UK; 5 Discipline of Physiotherapy, School of Health, 5333 University of the Sunshine Coast, QLD, Australia; 6 University of Canberra, Discipline of Sport and Exercise Science, Faculty of Health, University of Canberra, Canberra, ACT, Australia; 7 University of Canberra, Research Institute for Sport and Exercise, Canberra, ACT, Australia

**Keywords:** tennis elbow, outcome measures, assessment, management

## Abstract

**Objective:**

To examine the frequency of use and establish routine clinical practice of patient-reported outcome measures (PROMs), performance-based OMs and clinician-reported OMs in the assessment of a suspected case of lateral elbow tendinopathy (LET).

**Methods:**

Physiotherapists from eight countries completed an anonymous online survey, rating their frequency of use (never, rarely, sometimes, often and always) for unidimensional PROMs, multidimensional PROMs, performance-based OMs and clinician-reported OMs. To establish clinical practices, responses were dichotomised into routine (≥70% often/always) and not-routine (≥70% sometimes/rarely/never); items below both thresholds were classified as neither.

**Results:**

Two hundred ninety-nine respondents completed the survey. No outcome measure met the criteria for routine practice. Eight of 17 multidimensional PROMs, and six of eight clinician-reported OMs met the criteria for not-routine practice. All unidimensional PROMs and performance-based OMs, and a select number of multidimensional PROMs and clinician-reported OMs did not meet the threshold for routine or not-routine practice.

**Conclusions:**

Our results suggest no single outcome measure is routinely used by physiotherapists assessing a suspected case of LET. It is plausible that physiotherapists select OMs based on patient presentation, rather than the clinical diagnosis, or that outcome measures are perhaps seen more as a research tool than common place in clinical practice.

## Introduction

Lateral elbow tendinopathy (LET), colloquially known as tennis elbow, is a musculoskeletal condition affecting 1–3% of the general population,^1^ 12–25% of factory workers,^2^ and up to 40% of tennis players.^3^ It is characterised by pain provoked by gripping and object manipulation.^4^ Although LET is relatively straightforward to diagnose, its pathophysiology is complex, involving structural^5^ and sensorimotor changes,^6–9^ as well as psychosocial impacts such as social withdrawal, anxiety and reduced quality of life.^10,11^

Given this complexity, a person-centred care approach is essential, supported by the appropriate use of evidence-based outcome measures (OMs). Over 50 published OMs are available for LET,^12^ spanning several domains.^13,14^ Patient-reported outcome measures (PROMs) are often described as being unidimensional or multidimensional and aim to capture pain and functional impairment from the patient's perspective.^15,16^ Performance-based OMs, often referred to as physical outcome measures, offer reliable and objective assessments of function.^15^ Clinician-reported OMs reflect patient-relevant outcomes via proxy reports or clinical observations^14,17^ and can guide complex decisions, such as surgical referral. With a wide range of distinct yet sometimes overlapping tools,^18,19^ and barriers to implementation such as increased time requirements and knowledge of which OMs to use,^20^ physiotherapists must carefully consider when, what and how OMs are used when managing LET.

Recent clinical guidelines recommend using both unidimensional (e.g., numerical pain rating scale) and multidimensional PROMs (e.g., Patient Rated Tennis Elbow Evaluation (PRTEE) and the Disability of the Arm, Shoulder and Hand (DASH)).^21,22^ A comprehensive Delphi study involving clinicians, researchers and patients has also established a core outcome set for LET (COS-LET).^12^ While previous research has examined physiotherapists’ use of OMs,^23–25^ there has been limited insight into the specific contexts in which these measures are applied (e.g., pain at rest vs. pain during gripping), making comparisons with current guidelines and COS-LET difficult.

To assess physical impairment in LET, performance-based OMs such as pain-free and maximal grip strength are recommended,^21,22^ with pain-free grip strength also included as an interim measure in the COS-LET.^12^ However, only two studies have investigated performance-based OMs use by physiotherapists, with one published over two decades ago^24^ and another examining only pain-free grip strength.^23^ A broader evaluation of use of performance-based OMs is needed to understand whether clinical practice aligns with guidelines and the COS-LET.

Clinician-reported OMs, such as the Roles and Maudsley score and the Mayo Elbow Performance Index, are mentioned in guidelines but not recommended due to limited validation in LET populations.^21^ Their frequency of use in clinical practice remains unexplored and may offer insights for future training. Given the multidimensional nature of LET, the wide range of available outcome measures, and gaps in the literature, this study aims to examine the frequency and routine clinical use of outcome measures in LET assessment.

## Materials and methods

### Study design

An online, anonymous cross-sectional survey was completed by registered physiotherapists from Australia, Canada, Hong Kong, Ireland, New Zealand, South Africa, United Kingdom (UK) and the United States (US). Countries were selected as members of the International Federation of Orthopaedic Manual Physical Therapists with English as an official language. The study was approved by the Institutional Human Research Ethics Committee (Approval number: 0000022956) and informed consent was obtained at the start of the survey. The survey was reported using the checklist for reporting of survey studies (CROSS).^26^

### Survey development

The survey was developed through Qualtrics Version May 2021 (Qualtrics, Utah, USA), using previous surveys as a framework.^27,28^ The 66-item survey was developed as part of a larger project evaluating self-reported physiotherapy practices for the assessment and management of LET. The assessment and diagnosis portion of the survey has been previously published along with a copy of the full survey as supplementary material.^29^ Respondents answered demographic questions including age, gender, postgraduate qualifications, country, clinical experience, work setting and estimated monthly LET patient numbers. Regarding this study, respondents reported their frequency of use for unidimensional and multidimensional PROMs, performance-based OMs and clinician-reported OMs, when assessing a suspected case of LET (tennis elbow). The three questions included: (1) When assessing a patient with a suspected case of tennis elbow, how often do you use the following Patient Reported Outcome Measures (PROMs)? (2) When assessing a patient with a suspected case of LET (tennis elbow), how often do you use the following physical outcome measures for LET (tennis elbow)? and (3) When assessing a patient with a suspected case of tennis elbow, how often do you use the following Clinician Reported Outcome Measures?. Within this manuscript physical outcome measures are referred to as performance-based OMs. A comprehensive list of outcome measures was included in the survey based on previous literature in LET^30^ and the experiences of the research team. The frequency of use of these outcome measures was scored using a 5-point Likert scale (never, rarely, sometimes, often and always). Respondents were requested to select ‘never’ if they had not previously heard of the outcome measure(s).

### Recruitment and data collection

The recruitment and data collection methods have been previously published^29^ with the survey open from May 2021 to June 2022. In brief, recruitment was undertaken using social media groups (e.g., Facebook and Twitter), and with assistance from the New Zealand Physiotherapy Society. Three $100AUD random prizes were used to optimise responses and were drawn from respondents who provided contact details in a separate form at completion of the survey. The method of identifying artificial bot-generated responses and sample size statistics have been previously reported.^29^

### Data analysis

Data analysis was undertaken using Statistical Package for the Social Sciences (SPSS) v26 (IBM Corp, IL, USA) and Microsoft Excel (Microsoft Corporation, CA, USA). Demographic data are presented using descriptive statistics (e.g., mean (SD), median (IQR), and number (%)). Ordinal data from Likert scales are presented in table format. To analyse the frequency of outcome measure use, we applied a previously published method.^29^ A combined response rate of ≥70% for ‘often’ or ‘always’ was classified as *routine* practice, while a combined response rate of ≥70% for ‘sometimes’, ‘rarely’ or ‘never’ was classified as *not routine* practice. If neither threshold was met, the outcome measure was considered neither routine nor not routine.

## Results

### Survey response

In brief, 596 people clicked the survey link, 144 were excluded using screening questions, 22 failed to consent, 121 dropped out early, and 10 were deemed bot-generated responses, leaving 299 valid and complete responses.^29^ The median (IQR) time to complete the survey was 21.8 (14.3–37.2) minutes.

### Demographic characteristics

**Figure 1.**
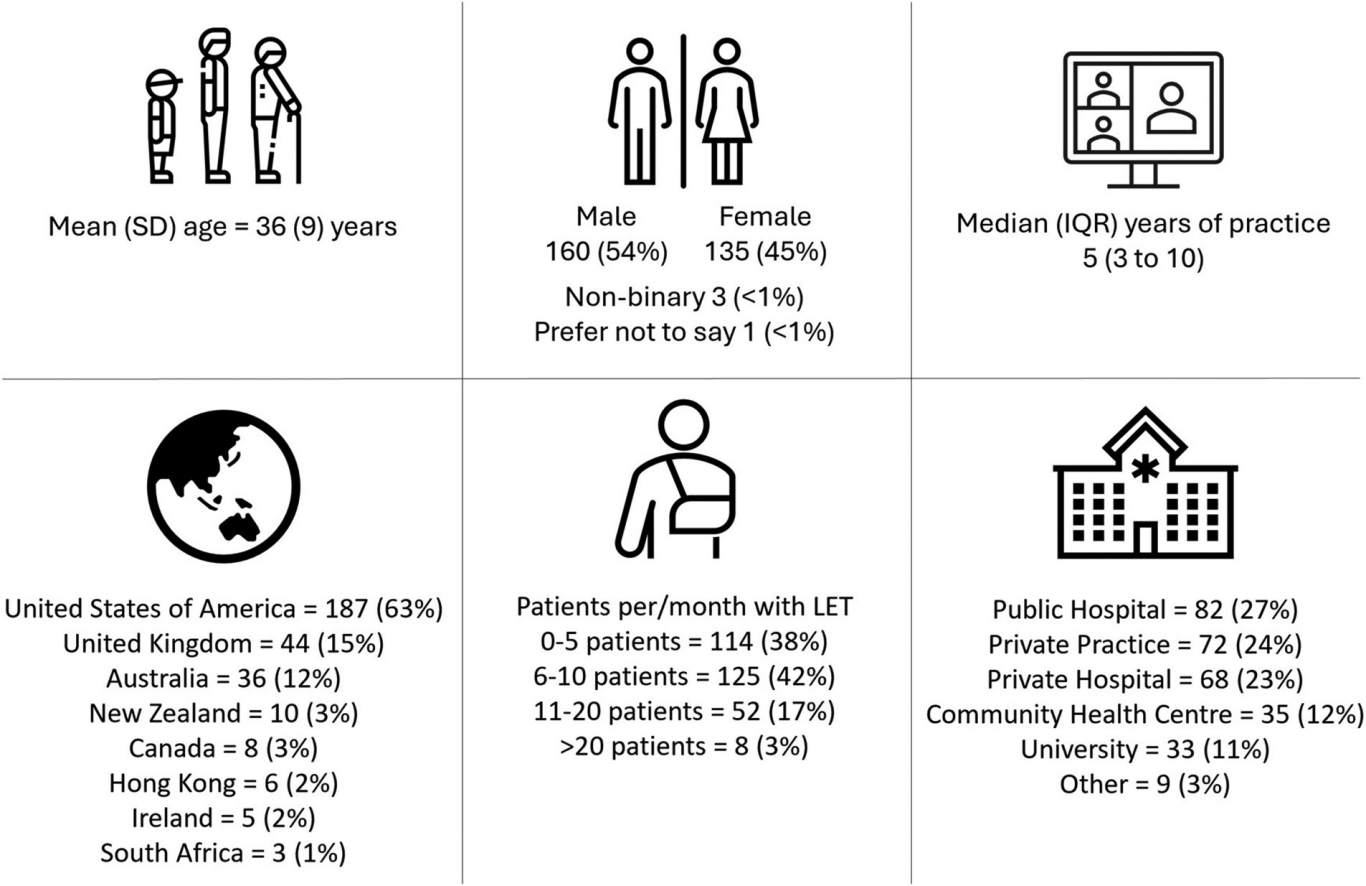
Respondent demographics. Data presented as *n* (%) of respondents, unless otherwise indicated. LET: lateral elbow tendinopathy.

Responder demographics have been previously reported^29^ and are provided in Figure 1. Approximately one third (30%, n = 91) did not have a postgraduate qualification. Of the 208 respondents with a postgraduate qualification, 23% (n = 48) had a postgraduate certificate or diploma, 43% (n = 90) had a Master of Physiotherapy or a Master of Philosophy, 35% (n = 72) had a Clinical Doctorate, 8% (n = 16) had a Doctor of Philosophy, and 22% (n = 46) had non-tertiary IFOMPT training.

### Use of outcome measures

**Figure 2.**
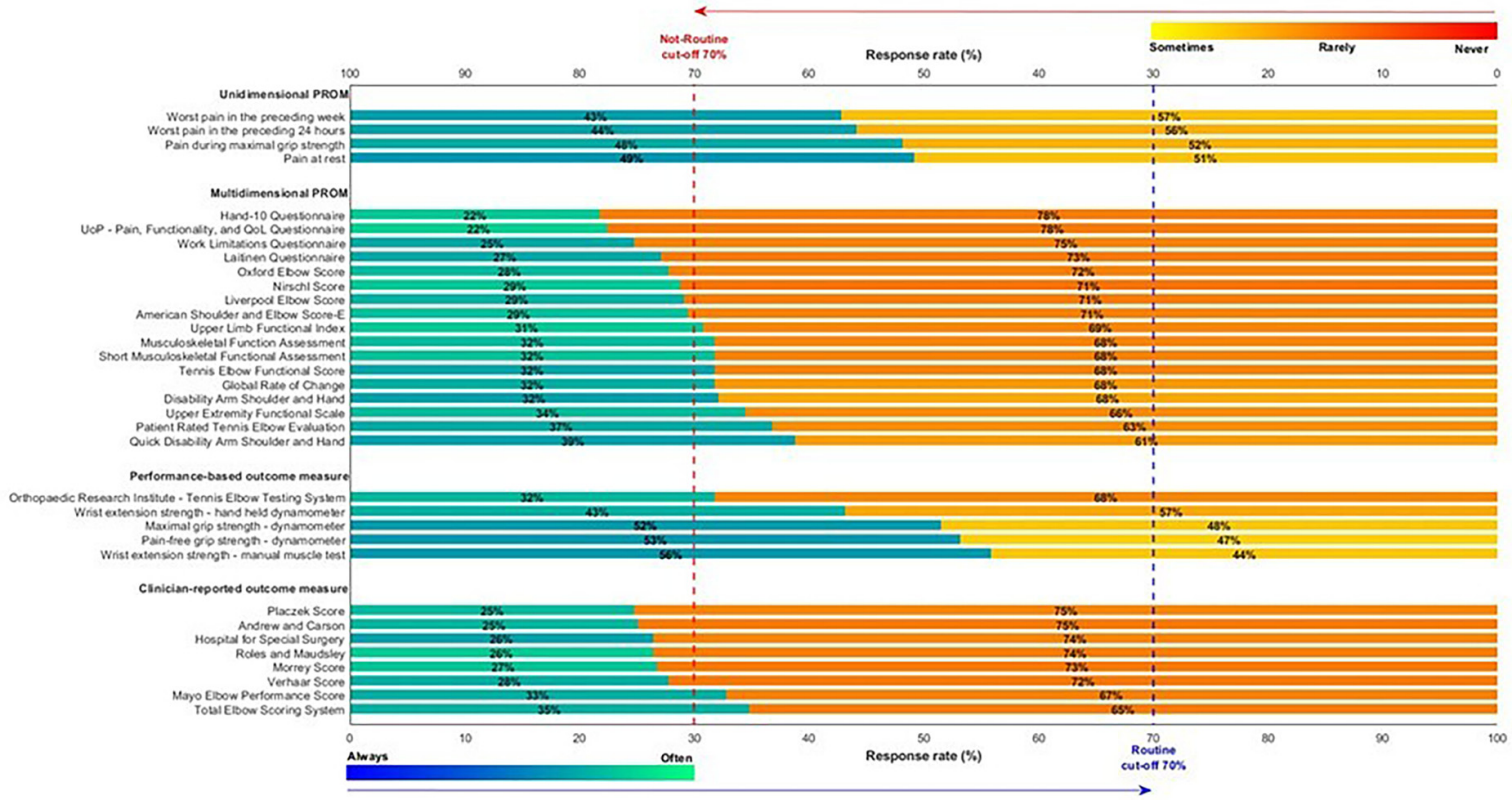
Horizontal stacked bar chart showing the reported frequency of use for physiotherapy outcome measures, grouped by conceptual category: unidimensional patient-reported outcome measures (PROM), multidimensional PROM, performance-based outcome measure and clinician-reported outcome measure. Each bar represents the proportion of respondents indicating “Routine” use (Always or Often; blue gradient) versus “Not routine” use (Sometimes, Rarely or Never; orange gradient). Percentages are displayed within each segment. Vertical dashed lines mark the 70% cut-off thresholds for routine (blue – bottom x-axis) and non-routine (red, top x-axis) use. Colour intensity within segments reflects response distribution.

Table 1 provides the frequency of use of each outcome measure. No outcome measure met the criteria for *routine* use based on the ≥70% threshold of ‘always and often’ used (Figure 2). Eight of 17 multidimensional PROMs met the criteria for *not-routine* use based on the ≥70% threshold of ‘sometimes, rarely, and never’ used (Figure 2). These outcomes included the Hand-10 Questionnaire (78%), University of Peloponnese Pain, Functionality, and Quality of Life Questionnaire (75%), Work Limitations Questionnaire (73%), Laitinen Questionnaire (72%), Oxford Elbow Score (72%), Nirschl Score (71%), Liverpool Elbow Score (71%), and American Shoulder and Elbow Score-E (71%). Six of eight clinical-reported OMs met the criteria for *not-routine* use including the Placzek Score (75%), Andrew and Carson (75%), Hospital for Special Surgery (74%), Roles and Maudsley (74%), Morrey Score (73%), and Verhaar Score (72%).

**Table 1.** The frequency of use of outcome measures in the assessment of a suspected case of lateral elbow tendinopathy.

Outcome measures	Always	Often	Sometimes	Rarely	Never
*Unidimensional PROMs*					
Pain at rest	59 (20%)	88 (29%)	96 (32%)	41 (14%)	15 (5%)
Pain during maximal grip	45 (15%)	99 (33%)	86 (29%)	44 (15%)	25 (8%)
Worst pain preceding 24 h	46 (15%)	86 (29%)	89 (30%)	54 (18%)	24 (8%)
Worst pain preceding week	46 (15%)	82 (27%)	110 (37%)	40 (13%)	21 (7%)
*Multidimensional PROMs*					
Quick Disability Arm Shoulder and Hand	42 (14%)	74 (25%)	86 (29%)	52 (17%)	45 (15%)
Patient Rated Tennis Elbow Evaluation	34 (11%)	76 (25%)	83 (28%)	44 (15%)	62 (21%)
Upper Extremity Functional Scale	25 (8%)	78 (26%)	84 (28%)	47 (16%)	65 (22%)
Disability Arm Shoulder and Hand	34 (11%)	62 (21%)	100 (33%)	67 (22%)	36 (12%)
Global Rate of Change	28 (9%)	67 (22%)	95 (32%)	54 (18%)	55 (18%)
Tennis Elbow Functional Score	32 (11%)	63 (21%)	83 (28%)	41 (14%)	80 (27%)
Short Musculoskeletal Functional Assessment	21 (7%)	74 (25%)	88 (29%)	34 (11%)	82 (27%)
Musculoskeletal Function Assessment	23 (8%)	72 (24%)	86 (29%)	40 (13%)	78 (26%)
Upper Limb Functional Index	18 (6%)	74 (25%)	85 (28%)	49 (16%0	73 (24%)
American Shoulder and Elbow Score-E	20 (7%)	68 (23%)	84 (28%)	45 (15%)	82 (27%)
Liverpool Elbow Score	25 (8%)	62 (21%)	80 (27%)	46 (15%)	86 (29%)
Nirschl Score	17 (6%)	69 (23%)	74 (25%)	48 (16%)	91 (30%)
Oxford Elbow Score	21 (7%)	62 (21%)	80 (27%)	59 (20%)	77 (26%)
Laitinen Questionnaire	26 (9%)	55 (18%)	78 (26%)	52 (17%)	88 (29%)
Work Limitations Questionnaire	24 (8%)	50 (17%)	88 (29%)	54 (18%)	83 (28%)
UoP - Pain, Functionality, and QoL Questionnaire	11 (4%)	56 (19%)	86 (29%)	51 (17%)	95 (32%)
Hand-10 Questionnaire	13 (4%)	52 (17%)	89 (30%)	51 (17%)	94 (31%)
*Performance-Based Outcomes*					
Wrist extension strength – MMT	64 (21%)	103 (34%)	82 (27%)	43 (14%)	7 (2%)
Pain-free grip strength – dynamometer	62 (21%)	97 (32%)	98 (33%)	33 (12%)	9 (4%)
Maximal grip strength - dynamometer	58 (19%)	96 (32%)	97 (32%)	35 (12%)	13 (4%)
Wrist extension strength – HHD	38 (13%)	91(30%)	90 (30%	44 (15%)	36 (12%)
ORI – Tennis Elbow Testing System	25 (8%)	70 (23%)	90 (30%)	35 (12%)	79 (26%)
*Clinician-Reported Outcomes*					
Total Elbow Scoring System	30 (10%)	74 (25%)	74 (25%)	40 (13%)	81 (27%)
Mayo Elbow Performance Score	29 (10%)	69 (23%)	84 (28%)	35 (12%)	82 (27%)
Verhaar Score	25 (8%)	58 (19%)	80 (27%)	46 (15%)	90 (30%)
Morrey Score	17 (6%)	63 (21%)	84 (28%)	44 (15%)	91 (30%)
Roles and Maudsley Score	15 (5%)	64 (21%)	78 (26%)	59 (20%)	83 (28%)
Hospital for Special Surgery	28 (9%)	51 (17%)	91 (30%)	46 (15%)	83 (28%)
Andrew and Carson	20 (7%)	55 (18%)	80 (27%)	55 (18%)	89 (30%)
Placzek Score	19 (6%)	55 (18%)	98 (33%)	40 (13%)	87 (29%)

Data reported as number (%). PROMs: Patient-reported outcome measure; UoP: University of Peloponnese; QoL: Quality of Life; MMT: manual muscle tests; HHD: hand-held dynamometer; ORI: Orthopaedic Research Institute.

## Discussion

Physiotherapists varied in their use of clinical OMs for suspected LET. Using the ≥70% ‘always’ or ‘often’ response rate threshold, no outcome measure met the criteria for *routine* practice. Although this may imply limited adherence to current clinical guidelines and the COS-LET^12,22,31,32^ or misalignment between guidelines and real-world practice, these guidelines were introduced during and after survey data collection, making consistent compliance unlikely. Rather than applying all outcome measures routinely, clinicians may be selecting tools based on evidence, clinical reasoning and/or patient-centred care, considering individual assessment findings and patient goals. Consistent with current clinical guidelines and the COS-LET, nearly half of the multidimensional PROMs (47%) and three-quarters of the clinician-reported OMs (75%) were classified as *not routine*, indicating infrequent use. These measures may be reserved for follow-up rather than initial assessment. Overall, the findings suggest that unidimensional PROMs and performance-based OMs are used more frequently, alongside a limited number of multidimensional PROMs and clinician-reported OMs, but none are used routinely.

### Unidimensional patient-reported outcome measures

No unidimensional PROMs met the threshold for *routine* or *not-routine* use, which may reflect the wide range of available measures and the varied clinical contexts in which they are applied. *Pain at rest* and *pain during gripping* were the most frequently used unidimensional PROMs yet still fell short of the *routine* use threshold. Only 5–8% of respondents reported ‘never’ using unidimensional PROMs for suspected LET, indicating pain scores are commonly but not routinely applied and may be contextualised through assessment stage, clinical reasoning, and/or patient-centred care. The use of unidimensional PROMs to some extent by 92–95% of our respondents appear consistent with current clinical guidelines which recommend the use of the numerical pain rating scale at baseline and at least one other timepoint.^21^ Similarly, our findings align with previous surveys reporting that 65–71% of healthcare professionals use the numerical pain rating scale for patients with LET.^23,24,33^ However, unlike previous surveys^23,24,33^ and current guidelines,^21^ which do not specify the context of use (e.g., pain at rest vs. during gripping), our results indicate that pain scores are applied across several different clinical contexts, including *pain at rest* and *pain during gripping*, and *pain across a specific timepoint*. In contrast, the COS-LET provides interim recommendations for using Items 1, 4 and 5 of the PRTEE to measure pain over a specified time, and pain during gripping rated using the numerical pain rating scale to measure pain on loading/activity.^12^ These interim recommendations were provided by the COS-LET due to a lack of clinometric research and validation of these specific outcome measures in the LET population at the time of publication.^12^ Recent evidence suggests that while pain on gripping is reliable in LET, it may lack responsiveness, making it unsuitable for tracking change over time.^34^ In contrast, the PRTEE pain subscale is reliable and responsive to change over time,^34^ making it a suitable outcome measure in the LET population. Consistent with current evidence, we recommend using the PRTEE pain subscale (Items 1, 4 and 5) to assess pain over time, with pain on gripping used to assess pain on loading until further evidence emerges. These recommendations are in line with our research findings, the COS LET and clinical guidelines, while practically also reducing the burden on patients and clinicians.

### Multidimensional patient-reported outcome measures

No multidimensional PROMs met the threshold for *routine* use, with nearly half (47%, 8 of 17) classified as *not-routine*, indicating infrequent application in suspected LET cases. The Quick-DASH and PRTEE were used most frequently, despite not reaching the threshold for *routine* use. Although our survey included more nuanced questions regarding frequency of use, the findings generally align with previous surveys of Greek and Italian physiotherapists, which reported PRTEE and DASH usage rates ranging from 13% to 38% and 7% to 58%, respectively, highlighting their limited uptake in clinical practice.^23,25^ This lack of routine use may not be unexpected as evidence shows several barriers to routine PROM usage in clinical practice, such as time limits, limited knowledge and/or confidence of OMs, and lack of organisational support,^20^ highlighting the importance of training in the appropriate selection and use of evidence-based OMs in LET. Current clinical guidelines suggest the inclusion of the PRTEE^21,22^ and the DASH^21^ at baseline and at least one follow-up timepoint. The COS-LET recommends the PRTEE as a core outcome to measure disability, and an interim outcome to measure function.^12^ Taken together, these findings suggest a gap between recommended use of multidimensional PROMs, such as the PRTEE and DASH, and their actual application in clinical practice, as reflected in both our survey data and previous international studies, noting guidelines were published after these surveys were closed.

A systematic review by Evans et al.^30^ rated the quality of the patient-centred outcomes in LET using the standardised Evaluating Measures of Patient-Reported Outcomes (EMPRO) tool, and identified that four multidimensional PROMs met the minimum criteria for recommend use: DASH, Quick-DASH, PRTEE and the Oxford Elbow Score.^30^ Although the DASH and PRTEE were frequently used by respondents, the Oxford Elbow Score met the threshold for *not-routine* use. Speculatively, this may be due to the Oxford Elbow Score being originally developed for post-surgical patients,^35^ a context not captured in our survey and unlikely to reflect routine use in the initial assessment of suspected LET. As the PRTEE is recommended in guidelines,^21^ included in the COS-LET,^12^ and shown to be reliable and sensitive to change,^34^ we advocate its routine use in the LET population to assess pain and function. Alternatively, the DASH (or Quick-DASH) could be used if required; for example, if there is no translated version of the PRTEE in the required language or if funding bodies require a more general assessment of the function of the upper limb.

### Performance-based outcome measures

None of our included performance-based OMs met our criteria for *routine* or *not-routine* use. However, manual muscle testing of the wrist extensors was the most frequently used, followed closely by pain-free grip strength and maximal grip strength, with only 2–4% of respondents reporting they ‘never’ use these three measures. Our findings are consistent with a previous survey which reported 65% of physiotherapists always or frequently test muscle strength, most commonly wrist extension, and 46% always or frequently use grip strength as an outcome measure for LET.^24^ Our findings and that of others^24^ appear not to align with current clinical guidelines which suggest there is little evidence to support the use of wrist extension strength as an outcome in those with LET.^21^ To assess functional capacity in LET, the COS-LET recommends pain-free grip strength over maximal grip strength as an interim performance-based OM,^12^ with recent evidence highlighting good clinometric properties of pain-free grip.^34^ While maximum grip strength may aid diagnosis,^36^ it often provokes pain, potentially affecting subsequent assessments,^8^ and is not sensitive to change over time.^34^ In contrast, pain-free grip strength is considered functional and non-provocative,^37^ reliable,^34,38^ sensitive to change over time^34,39^; and correlates with the PRTEE^34^ and self-perceived pain-free function,^40^ making it an important outcome measure in LET management.

### Clinician-reported outcome measures

Three quarters of clinician-reported OMs (75%, 6 of 8) met our criteria for *not-routine* use suggesting they are infrequently used, while no clinician-reported OM met the criteria for *routine* use. No previous studies of health professionals have reported the use of clinician-reported OMs in the management of LET. While current guidelines for LET mention the Mayo Elbow Performance Index and the Roles and Maudsley Score, these clinician-reported OMs are not recommended due to a lack of validation within the LET population.^21^ One possible reason for the low use of clinician-reported OMs may be that respondents were asked about their use in ‘suspected cases’ of LET. Since clinician-reported OMs are typically used to support complex decision-making, such as surgical suitability or post-operative outcomes, they may not be relevant during initial assessment. While tools like the Roles and Maudsley Score may be useful for tracking change after treatment, their application should be approached with caution due to limited validation in the LET population.

### Limitations

There are several limitations associated with survey-based research including, the inability to establish a causal relationship between current practice and practices recommended by clinical guidelines and the inability to confirm that anonymous respondents were registered physiotherapists.^29^ More specific to this study, there are other limitations that required consideration. First, given that current guidelines and the COS-LET^12,22,31,32^ were published during and after data collection of this survey, it would be unreasonable to expect consistent compliance with clinical recommendations, and these results likely provide a strong baseline for future research to examine whether publication of clinical guidelines alters clinical practice. Second, our survey did not include all possible outcome measures relevant to the management of chronic musculoskeletal pain and dysfunction, including measures of quality of life (e.g., EQ5D) or psychological factors (e.g., Tampa Scale of Kinesiophobia, Hospital Anxiety and Depression Scale). Third, while respondents were from eight different counties, our findings cannot be generalised to international practice due to the high proportion of respondents from the USA. Fourth, the high proportion of respondents with postgraduate qualifications may reflect participation from countries where such qualifications are required to enter the physiotherapy profession. Postgraduate-qualified individuals may be more engaged with current evidence and more likely to respond, potentially introducing bias. Finally, we did not include the Patient Specific Functional Scale (PSFS). Given the PSFS is recommended by guidelines,^21^ can be tailored to the individuals’ functional limitations,^41^ and is valid, reliable, and responsive to change in patients with upper limb musculoskeletal conditions,^42^ it is plausible that the PSFS is routinely used in the management of LET.

## Conclusion

This study highlights the variability in the frequency of outcome measure usage among physiotherapists managing suspected cases of LET. Although none of the outcome measures reported in this study met the criteria for *routine* use, which appears inconsistent with current clinical recommendations, expectation of compliance with guidelines is unlikely given the COS-LET and clinical guidelines were published during and after our survey data collection. These findings may therefore serve as an important baseline for future investigations. The fact that no outcome measure met the criteria for *routine* use, may suggest that clinicians use specific outcome measures based on patient-centred care, assessment stage, clinical reasoning and/or other practical constraints (e.g., time, availability). Greater alignment between guideline development, clinician education and real-world practice may be needed to support consistent and evidence-informed use of outcome measures in LET assessment.

## Supplemental Material

sj-docx-1-sel-10.1177_17585732261440542 - Supplemental material for Physiotherapists’ use of outcome measures in the assessment of lateral elbow tendinopathy: An international online survey Supplemental material, sj-docx-1-sel-10.1177_17585732261440542 for Physiotherapists’ use of outcome measures in the assessment of lateral elbow tendinopathy: An international online survey by Luke Heales, Leanne Bisset, Bill Vicenzino, Marcus Bateman, Caitlin Hill, Crystal Kean and Steven Obst in Shoulder & Elbow
